# Association between *Helicobacter pylori* infection and mortality risk in prostate cancer patients receiving androgen deprivation therapy: A real‐world evidence study

**DOI:** 10.1002/cam4.4318

**Published:** 2021-09-29

**Authors:** Jui‐Ming Liu, Chun‐Te Wu, Ren‐Jun Hsu, Wen‐Lin Hsu

**Affiliations:** ^1^ Division of Urology Department of Surgery Taoyuan General Hospital Ministry of Health and Welfare Taoyuan Taiwan; ^2^ Department of Obstetrics and Gynecology Tri‐Service General Hospital National Defense Medical Center Taipei Taiwan; ^3^ Department of Urology Chang Gung Memorial Hospital Keelung Taiwan; ^4^ Graduate Institute of Life Sciences National Defense Medical Center Taipei Taiwan; ^5^ Cancer Center Hualien Tzu Chi Hospital Buddhist Tzu Chi Medical Foundation Hualien Taiwan; ^6^ School of Medicine College of Medicine Tzu Chi University Hualien Taiwan; ^7^ Department of Radiation Oncology Hualien Tzu Chi Hospital Buddhist Tzu Chi Medical Foundation Hualien Taiwan

**Keywords:** androgen deprivation therapy, *Helicobacter* *pylori*, mortality, prostate cancer

## Abstract

**Purpose:**

*Helicobacter pylori* (*H. pylori*) is a major risk factor for gastric cancer and may affect androgen activity in men. The association between *H. pylori* and androgen deprivation therapy (ADT) in patients with prostate cancer (PCa) remains unclear.

**Methods:**

This retrospective cohort study linked National Health Insurance (NHI) data to Taiwan Cancer Registry (TCR) and Taiwan Death Registry (TDR) between 1995 and 2016. PCa patients who received ADT were classified into *H. pylori* infection and non‐*H. pylori* infection groups. The outcomes were overall mortality, prostate cancer‐specific mortality, and castration‐resistant prostate cancer (CRPC). Propensity score matching was adopted for the primary analysis and inverse probability of treatment weighting (IPTW) was used for the sensitivity analysis.

**Results:**

Of the 62,014 selected PCa patients, 23,701 received ADT, of whom 3516 had *H. pylori* infections and 20,185 did not. After matching, there were 3022 patients in the *H. pylori* infection group and 6044 patients in the non‐*H. pylori* infection group. The mean follow‐up period for the matched cohort was 4.8 years. Compared to the non*‐H*. *pylori* group, the *H. pylori* group was significantly associated with decreased risks of all‐cause mortality (hazard ratio [HR] 0.90; 95% confidence interval [CI] 0.84–0.96) and prostate cancer‐specific mortality (HR 0.88; 95% CI 0.81–0.95) in the matched analysis.

**Conclusions:**

*H. pylori* infection was associated with a reduced risk of mortality in PCa patients receiving ADT.

## 1 INTRODUCTION

*Helicobacter pylori* (*H. pylori*) is a gram‐negative curved bacillus that colonizes the stomach and that was first described in the 1980s.^1^ The gastric colonization of *H. pylori* is associated with gastric cancer.^2^ *H. pylori* is also associated with extra‐gastrointestinal diseases including cardiovascular disease, autoimmune disorders, skin disorders, and platelet disorders.^3^, ^4^

These extra‐gastrointestinal phenomena of *H. pylori* may occur due to the direct effects of the bacterium, the activation of inflammatory processes resulting from cytokine release, or the mimicry between bacterial and host antigens.^5^, ^6^ The prostate may also be affected by *H. pylori* as extra‐gastrointestinal infection.^7^

Eastern Asian populations have higher incidences of gastric cancer and lower incidences of prostate cancer (PCa) than Western countries.^8^, ^9^, ^10^ In addition, Eastern Asian populations have lower androgen‐related parameters, such as testicular weight, than Caucasians. The effects of *H. pylori* may be associated with the above observations. In the elderly, *H. pylori* could modulate androgen production.^11^ It has further been reported that *H. pylori* is associated with lower androgen activity in men.^12^ Lower androgen activity may, in turn, have some beneficial effects on PCa and androgen deprivation therapy (ADT).

Prior to the present study, however, a comprehensive analysis of the relationship between *H. pylori* infection and ADT for PCa had not been undertaken. We therefore conducted the first large‐scale real‐world evidence study utilizing data from the National Health Insurance program of Taiwan in order to determine whether *H. pylori* is associated with the outcomes of patients receiving ADT for PCa.

### 1.1 PATIENTS AND METHODS

#### 1.1.1 Data source

This retrospective cohort study linked National Health Insurance (NHI) data to Taiwan Cancer Registry (TCR) and Taiwan Death Registry (TDR) data using unique and de‐identified civil identification numbers. The NHI data were taken from a national health and welfare administrative database for Taiwan’s NHI program since 1995. The NHI data were collected by the National Health Informatics Project (NHIP) and managed by the Health and Welfare Data Science Center (HWDC). We analyzed the NHI, TCR, and TDR data from 1995 to 2016. Data for gender, birth date, medications, and diagnostic codes (the International Classification of Diseases, Ninth or Tenth Revision, Clinical Modification; ICD‐9‐CM or ICD‐10‐CM) were retrieved for the analyses performed in this study.

### 1.2 Study population

**FIGURE 1:**
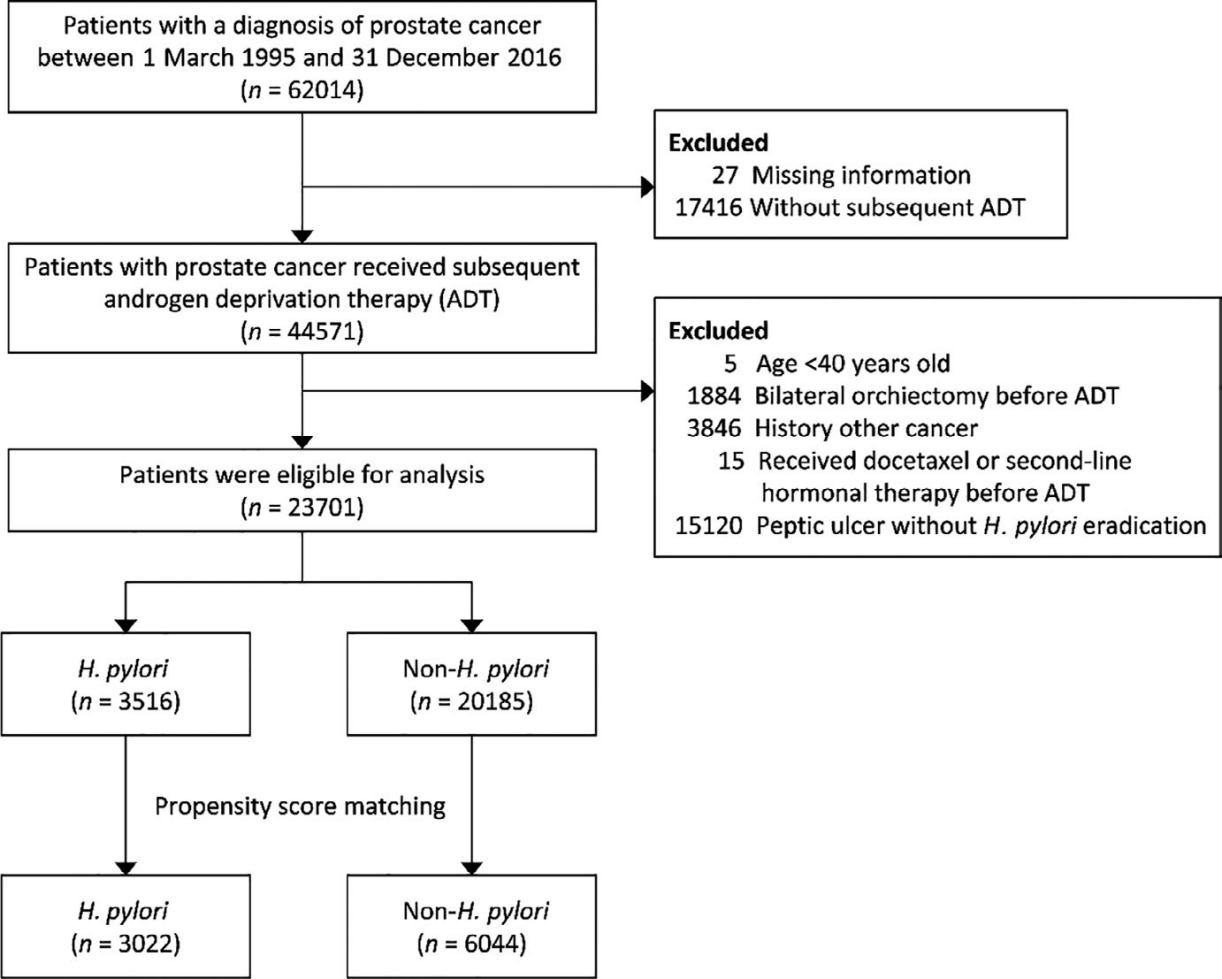
Patient selection

The patients with PCa in the TCR between March 1995 and December 2016 were identified using ICD‐9‐CM, ICD‐10‐CM, or ICD‐O‐3 (International Classification of Diseases for Oncology, Third Edition) diagnostic codes (Table S1). The patients with PCa who received subsequent ADT therapy were then identified. The ADT categories are detailed in the Supplementary Appendix. The use of ADT therapy was detected using the Taiwan NHI reimbursement codes, which can be referred to using the anatomic therapeutic chemical (ATC) classification system^13^ (Table S2). The date of receiving ADT therapy for PCa was defined as the index date. Patients who were less than 40 years old; had a cancer other than PCa; or received a bilateral orchiectomy, chemotherapy, abiraterone acetate, or enzalutamide were excluded. Finally, patients who had a history of peptic ulcer disease without subsequent eradication were excluded because the status of *H. pylori* infection for these patients was unknown (Figure 1).

### 1.3 Definition of exposure

The exposure of interest in this study was *H. pylori* infection. The *H. pylori* infection was defined as having a diagnosis of peptic ulcer and receiving subsequent eradication therapy before the index date. In Taiwan, *H. pylori* eradication consisting of treatment with a proton pump inhibitor (PPI) or histamine receptor‐2 blocker (H2 receptor blocker), clarithromycin or metronidazole, and amoxicillin or tetracycline with or without bismuth for a duration of 7–14 days is the standard treatment for *H. pylori* (Table S2). Patients were divided into two groups according to the status of *H. pylori* infection. Patients with *H. pylori* infection were further divided into two subgroups according to the timing between the initial diagnosis of peptic ulcer and the eradication therapy. Patients who received *H. pylori* eradication therapy within 1 year of the diagnosis date of peptic ulcer were classified as the early eradication subgroup, while the other patients were classified as the late eradication subgroup (Figure 1).^14^

### 1.4 Study outcomes

This study included three outcomes: all‐cause mortality, PCa‐specific mortality, and castration‐resistant prostate cancer (CRPC). The survival status, date of death, and cause of death of patients were verified in the TDR. PCa patients diagnosed with CRPC were defined as PCa patients who received chemotherapy, abiraterone acetate, or enzalutamide. The drugs used to treat patients were also identified using the Taiwan NHI reimbursement codes (Table S3). The end dates for follow‐up were extracted from the survival status in the TDR. Patients were followed until the date of death, event occurrence, or 31 December 2016, whichever came first.

### 1.5 Covariates

The covariates in this study consisted of potential confounders including age, urbanization level, monthly income, comorbidities, history of events, Charlson Comorbidity Index (CCI) score, PCa stage at diagnosis, disease extent, and medications. The demographics and socioeconomic information were included in the Registry for Beneficiaries, which is a sub‐database of the NHI database. Comorbidities at the index date were identified based on having two outpatient diagnoses or a single inpatient diagnosis in the previous year. The history of a given event (e.g., cerebrovascular disease) was identified based on a single inpatient diagnosis before the index date. The ICD‐9‐CM and ICD‐10‐CM diagnostic codes for the detection of diseases are listed in Table S1. The PCa stage at diagnosis was extracted from the TCR, while medication use in the year preceding the index date was also extracted.

### 1.6 Statistical analysis

To reduce confounding due to potential selection bias, a propensity score matching (PSM) method was employed as the primary analysis in this study. The propensity score was the predicted probability of being in the *H. pylori* group given the values of covariates using logistic regression. Each patient in the *H. pylori* group was matched with two counterparts in the non‐*H. pylori* group. The quality of matching was checked using the absolute value of the standardized difference (STD). As to the fatal time to event outcomes, the risks between the two groups were compared using the Cox proportional hazard model with hazard ratio (HR). The incidences of CRPC for the two groups were compared using the Fine and Gray subdistribution hazard model^15^, which considered all‐cause mortality as a competing risk. PSM and the same survival analyses were also performed to compare the early eradication subgroup with the late eradication subgroup. Subgroup analysis was performed to determine whether the effects of *H. pylori* infection on all‐cause death and PCa‐specific death were consistent among the different levels of the nine pre‐specified subgroup variables listed in the Supplementary Appendix.

Sensitivity analysis was performed to assess the robustness of the primary analysis. Propensity score matching causes some amount of data loss that would reduce the statistical power. Therefore, we used inverse probability of treatment weighting (IPTW) based on the propensity scores to estimate the average treatment effect of *H. pylori* infection without loss of patients in a simulated population. A two‐sided *p* value < 0.05 was considered to be statistically significant and no adjustment of multiple testing (multiplicity) was made in this study. All statistical analyses were performed using SAS version 9.4 (SAS Institute).

## 2 RESULTS

### 2.1 Patient characteristics

A total of 62,014 PCa patients were selected between 1995 and 2016, of whom 44,571 received subsequent ADT. After applying the exclusion criteria, there were 23,701 remaining patients, of whom 3516 had *H. pylori* infections and 20,185 did not. After matching, there were 3022 patients in the *H. pylori* infection group and 6044 patients in the non‐*H. pylori* infection group (Figure 1). In the matched cohort, a total of 4042 patients died, including 42.5% (1284/3022) and 45.6% (2758/6044) of the patients in the *H. pylori* infection group and the non*‐H. pylori* group, respectively. The mean follow‐up period for the matched cohort was 4.8 years (standard deviation = 3.2 years).

The demographic characteristics of the study subjects are listed in Table 1. Before matching, the patients in the *H. pylori* group had higher prevalence of hypertension, diabetes, coronary heart disease, hyperlipidemia, chronic obstructive pulmonary disease, chronic kidney disease, chronic liver disease, cerebrovascular disease, and higher CCI scores. The *H. pylori* group also had more distant metastases. In addition, more patients in the *H. pylori* group were prescribed non‐steroidal anti‐inflammatory drugs, clopidogrel, Cox‐2 inhibitors, oral hypoglycemic agents, and angiotensin‐converting enzyme inhibitor/angiotensin II receptor blockers. After matching, however, there were no differences between the two groups.

**TABLE 1:** Characteristics of the patients with and without *Helicobacter pylori* infection before and after propensity score matching

Variables	Before matching			After matching		
	*H. pylori* (n = 3516)	Non‐*H. pylori* (n = 20185)	STD	*H. pylori* (n = 3022)	Non‐*H. pylori* (n = 6044)	STD
Age (year)	74.4 ± 8.2	74.0 ± 8.4	0.056	74.2 ± 8.4	74.2 ± 8.4	–0.003
Age group						
40–49 years	12 (0.3)	104 (0.5)	–0.028	12 (0.4)	19 (0.3)	0.015
50–59 years	165 (4.7)	1180 (5.9)	–0.052	159 (5.3)	315 (5.2)	0.002
60–69 years	799 (22.7)	4698 (23.3)	–0.013	710 (23.5)	1428 (23.6)	–0.003
70–79 years	1605 (45.7)	9295 (46.1)	–0.008	1368 (45.3)	2720 (45.0)	0.005
≥80 years	935 (26.6)	4908 (24.3)	0.052	773 (25.6)	1562 (25.8)	–0.006
Urbanization						
1 (most urbanized)	757 (21.5)	4647 (23.0)	–0.036	656 (21.7)	1270 (21.0)	0.017
2	1387 (39.5)	7717 (38.2)	0.025	1186 (39.3)	2378 (39.3)	–0.002
3	902 (25.7)	5054 (25.0)	0.014	782 (25.9)	1586 (26.2)	–0.008
4 (least urbanized)	470 (13.4)	2767 (13.7)	–0.010	398 (13.2)	810 (13.4)	–0.007
Monthly income, NT$						
Dependent	5 (0.1)	22 (0.1)	0.008	5 (0.2)	6 (0.1)	0.019
<15,000	1304 (37.1)	7737 (38.3)	–0.026	1096 (36.3)	2184 (36.1)	0.003
15,000–24,999	1350 (38.4)	7814 (38.7)	–0.006	1176 (38.9)	2358 (39.0)	–0.002
≥25,000	857 (24.4)	4612 (22.9)	0.036	745 (24.7)	1496 (24.8)	–0.002
Comorbidity						
Hypertension	2102 (59.8)	8541 (42.3)	0.355	1698 (56.2)	3461 (57.3)	–0.022
Diabetes	813 (23.1)	3160 (15.7)	0.190	676 (22.4)	1360 (22.5)	–0.003
Coronary heart disease	795 (22.6)	2855 (14.1)	0.220	558 (18.5)	1122 (18.6)	–0.003
Hyperlipidemia	536 (15.2)	1687 (8.4)	0.214	403 (13.3)	800 (13.2)	0.003
Atrial fibrillation	115 (3.3)	487 (2.4)	0.052	97 (3.2)	204 (3.4)	–0.010
Peripheral arterial disease	80 (2.3)	273 (1.4)	0.070	62 (2.1)	117 (1.9)	0.008
COPD	518 (14.7)	2007 (9.9)	0.146	398 (13.2)	826 (13.7)	–0.015
Chronic kidney disease	477 (13.6)	1729 (8.6)	0.160	381 (12.6)	769 (12.7)	–0.003
Chronic liver disease	453 (12.9)	1146 (5.7)	0.250	302 (10.0)	554 (9.2)	0.028
Alcoholism	17 (0.5)	60 (0.3)	0.029	15 (0.5)	26 (0.4)	0.010
History of event						
Old myocardial infarction	101 (2.9)	336 (1.7)	0.081	75 (2.5)	126 (2.1)	0.027
Heart failure	151 (4.3)	513 (2.5)	0.096	112 (3.7)	243 (4.0)	–0.016
Cerebrovascular disease	351 (10.0)	1264 (6.3)	0.137	269 (8.9)	566 (9.4)	–0.016
CCI score	3.9 ± 2.5	2.9 ± 2.5	0.399	3.8 ± 2.6	3.7 ± 2.4	0.033
CCI score group						
0	95 (2.7)	3125 (15.5)	–0.456	95 (3.1)	189 (3.1)	0.001
1–2	955 (27.2)	8352 (41.4)	–0.303	946 (31.3)	1921 (31.8)	–0.010
≥3	2466 (70.1)	8708 (43.1)	0.566	1981 (65.6)	3934 (65.1)	0.010
Prostate cancer stage at diagnosis						
I	107 (3.0)	239 (1.2)	0.130	71 (2.4)	149 (2.5)	–0.008
II	801 (22.8)	2376 (11.8)	0.294	631 (20.9)	1228 (20.3)	0.014
III	417 (11.9)	1358 (6.7)	0.177	353 (11.7)	702 (11.6)	0.002
IV	737 (21.0)	2932 (14.5)	0.169	661 (21.9)	1346 (22.3)	–0.010
Unknown	1454 (41.4)	13280 (65.8)	–0.505	1306 (43.2)	2619 (43.3)	–0.002
Disease extent at ADT						
Locoregional	2617 (74.4)	16066 (79.6)	–0.123	2205 (73.0)	4402 (72.8)	0.003
Distant metastasis	899 (25.6)	4119 (20.4)	0.123	817 (27.0)	1642 (27.2)	–0.003
Medication						
NSAID	401 (11.4)	1424 (7.1)	0.151	290 (9.6)	592 (9.8)	–0.006
Aspirin	885 (25.2)	4735 (23.5)	0.040	787 (26.0)	1569 (26.0)	0.002
Clopidogrel	272 (7.7)	351 (1.7)	0.285	89 (3.0)	175 (2.9)	0.003
Cox‐2 inhibitor	219 (6.2)	450 (2.2)	0.200	108 (3.6)	213 (3.5)	0.003
Anticoagulant agents	70 (2.0)	322 (1.6)	0.029	60 (2.0)	117 (1.9)	0.004
Oral hypoglycemic agents	1058 (30.1)	4623 (22.9)	0.163	875 (29.0)	1786 (29.6)	–0.013
Insulin	101 (2.9)	402 (2.0)	0.057	84 (2.8)	184 (3.0)	–0.015
ACEI/ARB	1408 (40.1)	5611 (27.8)	0.261	1119 (37.0)	2276 (37.7)	–0.013
Follow‐up years	4.6 ± 3.1	5.7 ± 4.2	–0.298	4.8 ± 3.2	4.7 ± 3.1	0.054

STD, standardized difference; COPD, chronic obstructive pulmonary disease CCI, Charlson Comorbidity Index; ADT, androgen deprivation therapy; NSAID, non‐steroidal anti‐inflammatory drug; ACEI, angiotensin‐converting enzyme inhibitor; ARB, angiotensin II receptor blockers; GnRH, gonadotropin‐releasing hormone.

### 2.2 All‐cause mortality and prostate cancer‐specific mortality

**FIGURE 2:**
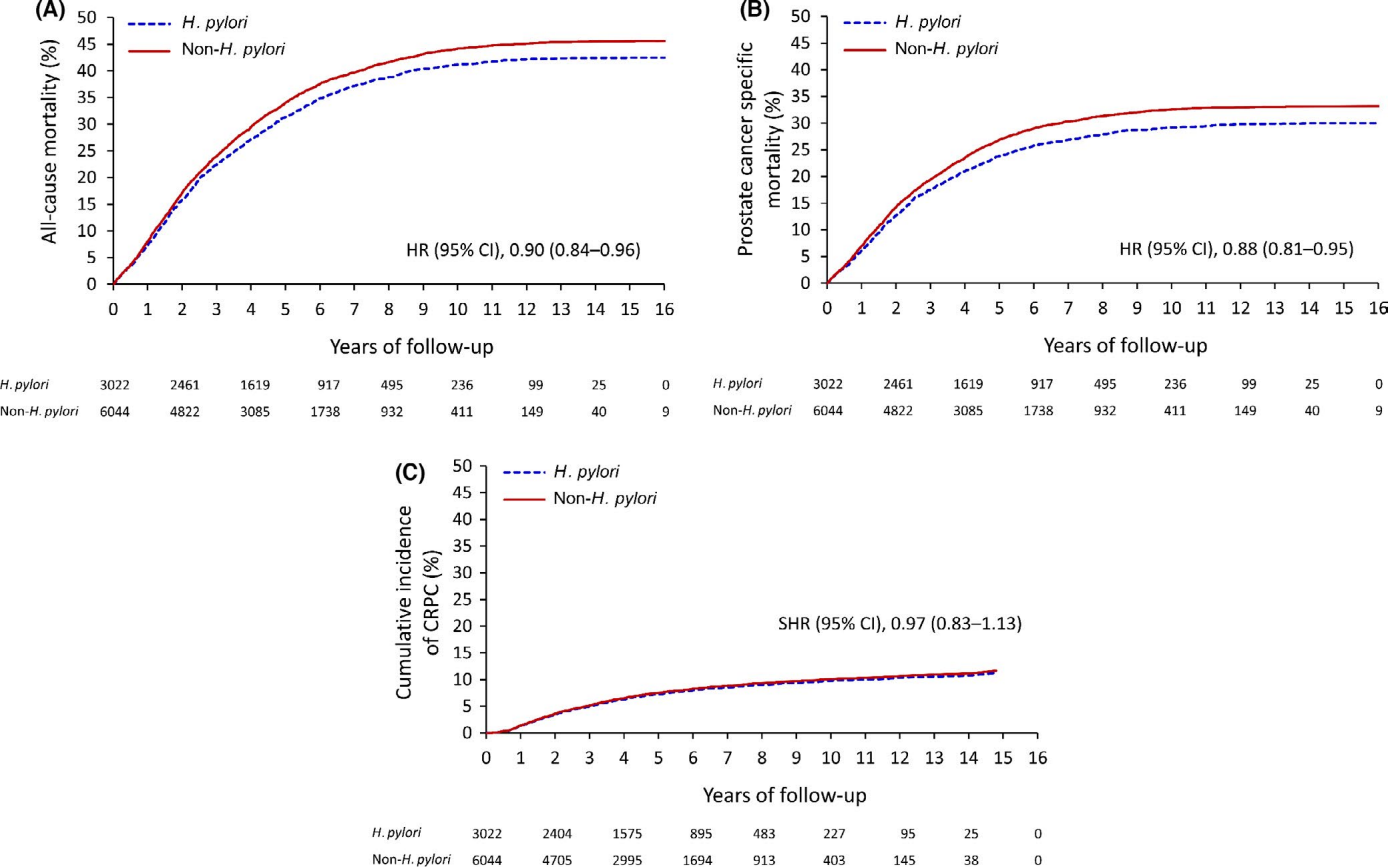
The unadjusted cumulative event rates of all‐cause mortality (A), prostate cancer‐specific mortality (B), and cumulative incidence function of castration‐resistant prostate cancer (C) of the patients with and without *H. pylori* infection in the propensity score matched cohort

The clinical outcomes of the PCa patients who received ADT in the *H. pylori* infection group and the non*‐H*. *pylori* group are shown in Table 2. Compared to the non*‐H*. *pylori* group, the *H. pylori* group was significantly associated with decreased risks of all‐cause mortality (HR 0.90; 95% CI 0.84–0.96) and PCa‐specific mortality (HR 0.88; 95% CI 0.81–0.95). The decreased risks of all‐cause mortality and PCa‐specific mortality in the *H. pylori* infection group were still observed in the sensitivity analysis by IPTW (Table S4). However, the risk of CRPC was not significantly different between the two groups (Table 2). The unadjusted cumulative event rates of all‐cause mortality and PCa‐specific mortality and the cumulative incidence rates of CRPC are depicted in Figure 2.

**TABLE 2:** Follow‐up outcomes of the patients with and without *Helicobacter pylori* infection

Outcomes	*H. pylori*		Non‐*H. pylori*		HR of *H. pylori* (95% CI)
	Event (%)	Incidence density	Event (%)	Incidence density	
Primary analysis: PSM					
All‐cause mortality	1284 (42.5)	8.81 (8.33–9.29)	2758 (45.6)	9.80 (9.43–10.16)	0.90 (0.84–0.96)
Prostate cancer‐specific mortality	906 (30.0)	6.21 (5.81–6.62)	2006 (33.2)	7.13 (6.82–7.44)	0.88 (0.81–0.95)
CRPC^b^	234 (7.7)	1.64 (1.43–1.85)	482 (8.0)	1.75 (1.59–1.90)	0.97 (0.83–1.13)
Sensitivity analysis: IPTW^a^					
All‐cause mortality	50.9%	9.18 (9.01–9.35)	57.1%	10.27 (10.10–10.44)	0.90 (0.81–0.99)
Prostate cancer‐specific mortality	32.8%	5.91 (5.77–6.04)	40.2%	7.23 (7.08–7.37)	0.81 (0.72–0.92)
CRPC^b^	8.4%	1.54 (1.47–1.61)	7.6%	1.39 (1.33–1.46)	1.13 (0.91–1.39)

HR, hazard ratio; CI, confidence interval; PSM, propensity score matching; IPTW, inverse probability of treatment weighting; CRPC, castration‐resistant prostate cancer.

^a^ Event values are given as %.

^b^ Estimated using the subdistribution hazard model which considered all‐cause death as a competing risk.

### 2.3 Subgroup analysis according to *H. pylori* eradication status

**FIGURE 3:**
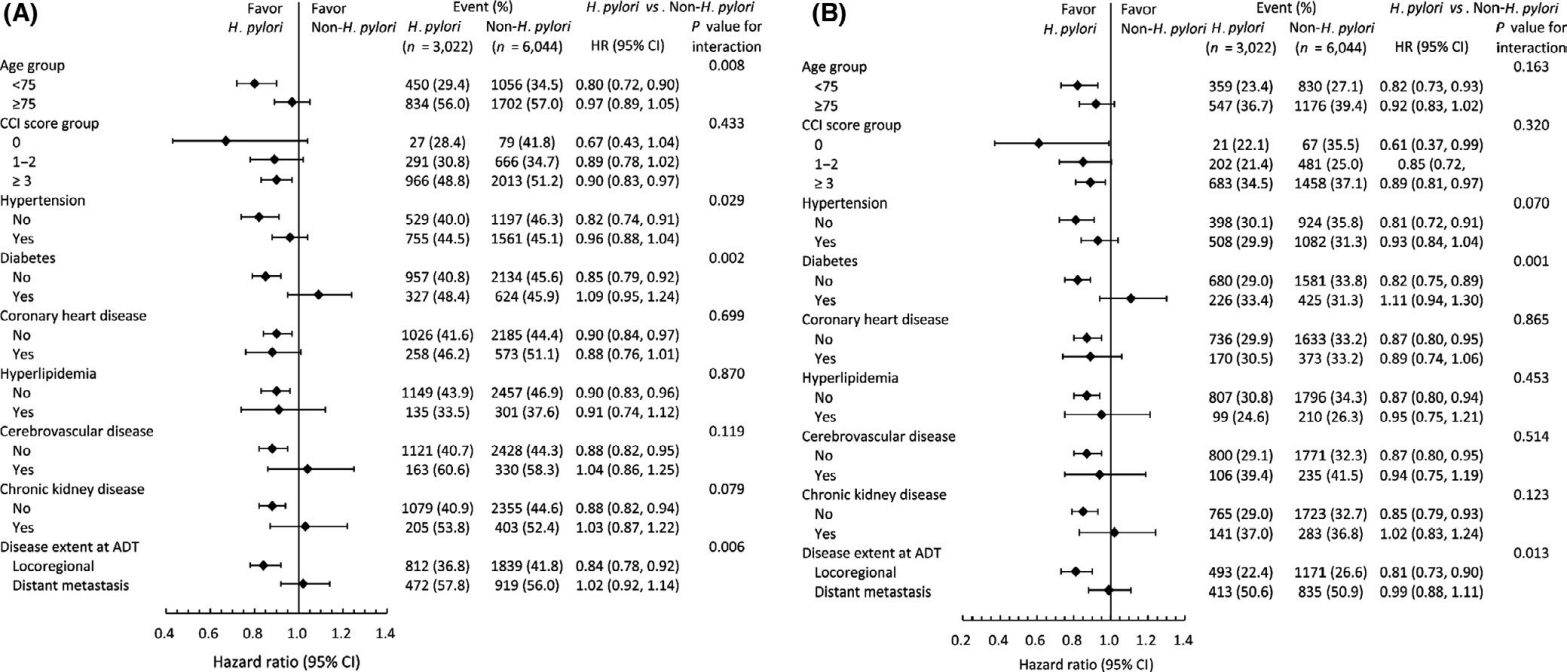
Pre‐specified subgroup analysis of all‐cause mortality (A) and prostate cancer‐specific mortality (B) in the propensity score matched cohort

The pre‐specified subgroup analysis results of all‐cause mortality and PCa‐specific mortality are shown in Figure 3. The observed decreased mortality risk from *H. pylori* infection was more apparent in patients who were younger than 75 years old, without hypertension, without diabetes, and who had locoregional PCa (*p *< 0.05) (Figure 3A). The observed decreased PCa‐specific mortality risk was more obvious in patients who did not have diabetes and locoregional PCa (*p *< 0.05) (Figure 3B).

We further compared outcomes in patients with *H. pylori* infection between the early eradication and late eradication groups. The characteristics of the patients with early eradication or late eradication of *H. pylori* are shown in Table S5 (PSM) and Table S6 (IPTW) in the Supplement. The results showed that there were no significant differences in all‐cause mortality, PCa‐specific mortality, and CRPC between the early eradication and late eradication subgroups (Table S7 and Figure S1).

## 3 DISCUSSION

To the best of our knowledge, this is the first real‐world evidence cohort study to investigate the association between *H. pylori* infection and mortality in PCa patients receiving ADT. Using PSM analysis on 62,014 PCa patients, we demonstrated that *H. pylori* infection was associated with lower risks of all‐cause mortality and PCa‐specific mortality in patients receiving ADT after adjustments for age, disease extent of PCa, comorbidities, and related medications. In addition, patients with early *H. pylori* eradication showed no statistically significant difference in mortality compared with those with late eradication. The results for 3022 *H. pylori* infection ADT patients and 6044 matched controls provide robust real‐world information that should prompt physicians to be aware of the potential protective effect of *H. pylori* infection in patients treated with ADT.

There are several possible mechanisms that might explain why *H. pylori* infection reduces mortality in PCa patients receiving ADT. *H. pylori* infection may have result in a changed human microbiome environment. Gut microbiota may be able to regulate sex hormones.^11^ It has been reported, for example, that changing the gut microbial environment in mice changes mice testosterone levels.^11^ Hosoda et al. reported that *H. pylori* absorbs and glucosylates androgens as membrane lipid components.^16^ In addition, *H. pylori* was found to be associated with lower androgen activity levels and lower levels of androstanediol glucuronide (3‐alphadiol‐G) (AAG), a testosterone biomarker, in older men in the NHANES III.^11^ *H. pylori* changes the gut microbiome, which may lead it to metabolize sex steroid hormones and influence their activity.^17^ *H. pylori* also reduces androgen levels, which may have a potential benefit to ADT for PCa. Chinese populations have high incidences of gastric cancer and lower incidences of PCa than Caucasians.^18^ Meanwhile, Chinese populations also have lower androgen‐related parameters, such as testicular weight, than Caucasians.^19^ The association of *H. pylori* with lower androgen levels may explain the above observations. Further studies are warranted, however, to achieve a better understanding of the effects of *H. pylori* infection.

Regimens for the eradication of *H. pylori* may affect androgen levels. PPIs, such as omeprazole, esomeprazole, or lansoprazole, may induce hepatic cytochrome P450 (CYP450), which increases testosterone metabolism and results, in turn, in reduced circulating testosterone levels.^20^, ^21^ H2 blockers, such as cimetidine, also have effects on CYP450, including further antiandrogen effects.^22^ Metronidazole has been reported to reduce androgen and cause gynecomastia.^23^ The antiandrogen effects of these regimens may have some benefit in ADT for patients with PCa. In a recent study, PPI inhibited apoptosis and further increased survival in hormone‐sensitive LNCaP cells via the ErbB2, MAPK‐ERK1/2, PI3K/Akt, and GSK‐3β pathways.^24^ Further studies are warranted, however, to elucidate the relationships between these regimens and ADT for PCa.

Meanwhile, *H. pylori* has extra‐gastrointestinal phenomena, with the prostate being a possible extra‐gastrointestinal colonization site.^7^ Immune responses evoked by *H. pylori* infection may be associated with chronic prostatitis.^7^, ^25^ Proliferative inflammatory atrophy (PIA) could be induced by prostatitis, and PIA is a suspected risk factor lesion for PCa development.^26^, ^27^ *H. pylori* may stimulate chronic systemic inflammatory responses through the production of various pro‐inflammatory cytokines, such as tumor necrosis factor‐α (TNF‐α), interferon‐γ (IFN‐γ), interleukin‐1β (IL‐1β), interleukin‐6 (IL‐6), and interleukin‐8 (IL‐8).^28^, ^29^ These pro‐inflammatory cytokines may in turn be associated with aggressive PCa types.^30^, ^31^, ^32^ On the other hand, higher regulatory T cell (Tregs) expression levels in patents with *H. pylori* colonization have been reported.^33^ Tregs may downregulate immune responses via TGF‐β or IL‐10 to reduce inflammatory responses.^34^, ^35^ Further studies are warranted, however, to elucidate the relationships between the cytokine effects of PCa and *H. pylori*.

The strength of this study is a large real‐world evidence study that enrolled 62,014 PCa patients with a long follow‐up period. We used the PSM analysis and IPTW analysis to ensure the accuracy of the study outcomes. The study included 3022 study subjects with *H. pylori* infection, with the risks of all‐cause mortality and PCa‐specific mortality being compared among those subjects and a matched control group. Subgroup analysis with age, hypertension, diabetes, hyperlipidemia, coronary heart disease, cerebrovascular disease, chronic kidney disease, CCI score, and disease extent of PCa was also conducted to investigate the effects of *H. pylori* infection on the risk of PCa mortality in order to provide substantial information for physicians.

Our study has several limitations. First, the PSA levels and Gleason scores were not available in the investigated databases. It was thus difficult to further evaluate the PCa of the included subjects. In addition, several potential confounding factors, including body mass index, educational level, intestinal microbiota, and inflammation parameters were not available in the investigated databases. Second, all the diagnoses were made using the ICD‐9, ICD‐10, or ICD‐O‐3 coding systems, such that diagnostic pitfalls may have occurred. Third, the NHI database is an administrative database, such that patients’ socioeconomic status, family history, personal health behaviors, laboratory data, and image findings were not available. We did not have data to confirm the exact duration of *H. pylori* infection in each study subject, and the severity of *H. pylori* was also not available in this study. Fourth, there were no data in the investigated databases to confirm whether early or late *H. pylori* eradication was successful. Finally, this population‐based study was a retrospective study, the association between *H. pylori*, intestinal microbiota, and prostate cancer may be lack of solid collateral evidence. To achieve a better understanding of the effects of *H. pylori* infection, further prospective studies are warranted to investigate the relationship between PCa mortality and *H. pylori* in patients with ADT.

## 4 CONCLUSIONS

This is the first real‐world evidence study to demonstrate that *H. pylori* infection is associated with a reduced risk of mortality in PCa patients receiving ADT. This novel finding provides clinical evidence of a potential role for *H. pylori* infection in ADT for PCa.

## AUTHORSHIP CONTRIBUTION

Liu JM and Hsu RJ thought of the concept and designed the study. Wu CT provided study materials. All authors collected and assembled data, conducted data analysis, wrote and made final approval of the manuscript.

## ETHICS STATEMENT

This study was approved by the Institutional Review Board of the Tri‐Service General Hospital (approval number: TSGHIRB No B‐107‐04). As this was a retrospective study and all data were anonymous, the Institutional Review Board department agreed with the authors that it was waived the need for informed consent.

## CONFLICT OF INTEREST

All authors declare no conflict of interest.

## Supporting information

Supplementary Material

## Data Availability

The datasets used and analyzed during the current study are not available for the restriction by the Institutional Review Board of the Tri‐Service General Hospital.
