# Supplementary material for: Association between Helicobacter pylori infection and mortality risk in prostate cancer patients receiving androgen deprivation therapy: A real‐world evidence study
Source: Cancer Med. 2021 Sep 29;10(22):8162–71. doi: 10.1002/cam4.4318 (PMC8607261; doi:10.1002/cam4.4318)
Supplement: Supplementary file 1 — Supplementary Material [file CAM4-10-8162-s001.docx]

**Supplementary File**

*Helicobacter pylori* Infection May Reduce the Risk of Mortality in Prostate Cancer Patients with Androgen Deprivation Therapy: A Real-World Evidence Study

We provided the supplementary file for additional information about this study

**Table of Contents**

Cover… 1

[Table of Contents 2](#_TOC_250009)

[Lists of Abbreviations and Terms](#_TOC_250007) 3

[Methods](#_TOC_250007) 4

1. Data source 4
2. [Definition of exposure 4](#_TOC_250006)
3. [Androgen deprivation therapy 5](#_TOC_250005)
4. [Covairates 5](#_TOC_250004)
5. [Statistical analysis 6](#_TOC_250003)

Supplementary Information 7

[Supplementary Table & Figure 8](#_TOC_250002)

1. [Table 1](#_TOC_250002) 8
2. [Table 2](#_TOC_250001)  9
3. [Table 3 1](#_TOC_250000)0
4. [Table 4 1](#_TOC_250000)1
5. [Table 5 1](#_TOC_250000)4
6. [Table 6 1](#_TOC_250000)7
7. [Table 7](#_TOC_250000) 21
8. [Figure 1A](#_TOC_250000) 22
9. [Figure 1B](#_TOC_250000) 23
10. [Figure 1C](#_TOC_250000) 24

[References 25](#_TOC_250002)

**Abbreviations**

AAG = androstanediol glucuronide (3-alphadiol-G)

ACEI = angiotensin converting enzyme inhibitor

ADT = androgen deprivation therapy

ARB = angiotensin II receptor blockers

ATC = anatomic therapeutic chemical

CCI = Charlson Comorbidity Index

CI = confidence intervals

CRPC = castration-resistant prostate cancer

CYP450 = cytochromes P450

GnRH = gonadotropin-releasing hormone

*H. pylori = Helicobacter pylori*

H2RA = histamine receptor-2 blocker

HR = hazard ratio

HWDC = Health and Welfare Data Science Center

ICD-9-CM = International Classification of Diseases, 9th revision, Clinical Modification

ICD-10-CM = International Classification of Diseases, 10th revision, Clinical Modification

ICD-O-3 = International Classification of Diseases for Oncology, Third Edition

IFN = interferon

IPTW = inverse probability of treatment weighting

KM = Kaplan-Meier

NHI = National Health Insurance

PCa = prostate cancer

PIA = proliferative inflammatory atrophy

PPI = proton pump inhibitor

PSA = prostatic specific antigen

PSM = propensity score matching

STD = standardized difference

TCR = Taiwan Cancer Registry

TDR = Taiwan Death Registry

Th = T helper (Th) cells

TNF = tumor necrosis factor

Tregs = regulatory T cells

**Methods**

**Data Source**

This retrospective cohort study was linked National Health Insurance (NHI) data with Taiwan Cancer Registry (TCR) and Taiwan Death Registry (TDR) by unique and de-identified civil identification number. The NHI data is a national health administrative database from Taiwan NHI program which is a universal health insurance system of Taiwan since 1995. The NHI program began in 1995 and covered more than 99% of the 23 million residents of Taiwan as of the end of 2013.[1]The NHI Data were collected by National Health Informatics Project (NHIP) and managed by Health and Welfare Data Science Center (HWDC). The NHI program covered approximately 99% of Taiwan’s 23.5 million residents for their complete outpatient visits, hospitalization records, prescription details and disease status since 1995. The TCR data included cancer site, behavior, diagnosis date, stage, treatment program, and recurrence information since 1979. For mortality and prostate cancer-specific mortality were primary outcomes of this study, the NHI data were further linked to the Taiwan Death Registry (TDR) which contained mandatory registered date, causes of death since 1971. Considering to different periods between the NHI, TCR and TDR data, we analyzed data from 1995 to 2016. Data for gender, birth date, medications, and diagnostic codes (based on the International Classification of Diseases, Ninth or Tenth Revision, Clinical Modification; ICD-9-CM or ICD-10-CM) [2,3]were retrieved for the analyses performed in this study.

**Definition of Exposure**

The exposure of interest in this study was *H. pylori* infection. The *H. pylori* infection was defined as having a diagnosis of peptic ulcer and receiving subsequent eradication therapy before the index date. According to the insurance benefit package of the NHI system, *H. pylori*-positive peptic ulcers are confirmed with gastroscopy and biopsy, endoscopy with a rapid urease test, histologic examination, microbial culture, and *helicobacter pylori* C13-breath test.[4]

**Androgen deprivation therapy**

Patients with prostate cancer (PCa) who received subsequent ADT therapy were included in this study. Androgen deprivation therapy (ADT) category was defined as the use of GnRH agonists (leuprolide, goserelin, and triptorelin), oral antiandrogens (cyproterone acetate, bicalutimide, and flutamide), and estrogens (diethylstilbestrol and estramustine).

**Covariates**

PCa stage at diagnosis was extracted from the TCR, while approximately half of this information were missing because the registration of PCa stage was mandatory since 2008. Information regarding the type of drug prescribed, date of prescription, days of supply, and total number of drug pills dispensed from pharmacy prescription database were collected. We selected potential covariates from NHI data, (TCR) and TDR including age, urbanization level, monthly income, comorbidities, history of events, the Charlson Comorbidity Index (CCI) score, PCa stage at diagnosis, disease extent and medications. The ICD-9-CM and ICD-10-CM diagnostic codes for comorbidities were listed in **Supplementary Table 1**.

**Statistical Analysis**

To reduce confounding due to potential selection-bias, a propensity-score matching (PSM) method was performed. The matching was processed using a greedy nearest neighbor algorithm with a caliper of 0.2 times of the standard deviation of the logit of propensity score, with random matching order and without replacement. The quality of matching was checked using the absolute value of standardized difference (STD) between the groups, where a value less than 0.1 was considered negligible difference.[5]

The propensity score was the predicted probability to be in the *H. pylori* group given values of covariates. The covariates selected to calculate propensity score were age, urbanization, monthly income, hypertension, diabetes, coronary heart disease, hyperlipidemia, atrial fibrillation, peripheral arterial disease, COPD, chronic kidney disease, chronic liver disease, alcoholism, old myocardial infarction, heart failure, cerebrovascular disease, Charlson comorbidity index (CCI) score, prostate cancer stage at diagnosis, disease extent of PCa, medication including NSAID, aspirin, clopidogrel, cox-2 inhibitor, anticoagulant agents, oral hypoglycemic agent, insulin, and ACEI / ARB.

The study group (*H. pylori* vs. non-*H. pylori*) was the only explanatory variable in the survival analyses. The within-pair clustering of outcomes after PSM was accounted for by using a robust standard error which was known as a marginal model. Subgroup analysis was performed to determine whether the effect of *H. pylori* infection on all-cause death and PCa specific death was consistent among different levels of the nine pre-specified subgroup variables, including age, CCI scores, hypertension, diabetes, coronary heart disease, hyperlipidemia, cerebrovascular disease, chronic kidney disease and PCa disease extent.

All statistical analyses were performed using SAS version 9.4 (SAS Institute, Cary, NC), including the procedures of ‘psmatch’ for propensity score matching, ‘phreg’ for survival analysis and the macro of ‘%cif’ for generating cumulative incidence function under the Fine and Gray subdistribution hazard method.[6]

**Information**

The ICD-9-CM and ICD-10-CM diagnostic codes for detection of diseases including study outcome and comorbidities in this study were detail listed in **Supplementary Table 1**. In Taiwan, *H. pylori* eradication consisting of proton pump inhibitor (PPI) or histamine receptor-2 blocker (H2 receptor blocker), clarithromycin or metronidazole, and amoxicillin or tetracycline with or without Bismuth as standard treatment of *H. pylori* with a duration of 7 to 14 days. Detail *H. pylori* eradication regimens are demonstrated in **Supplementary Table 2**. The detail list with ATC codes of pharmacotherapy in this study was demonstrated in **Supplementary Table 3**. Characteristics of the patients with and without *H. pylori* infection before and after inverse probability treatment weight was listed in **Supplementary Table 4**. Characteristics of the *H. pylori* infection patients with early or late-eradication before and after propensity score matching was listed in **Supplementary Table 5**. Characteristics of the H. pylori infection patients with early or late-eradication before and after inverse probability treatment weight was listed in **Supplementary Table 6**. Follow-up outcomes of the H. pylori infection patients with early or late-eradication was listed in **Supplementary Table 7**.

**Supplementary Figure 1**. The unadjusted cumulative event rates of all-cause mortality (A), prostate cancer-specific mortality (B) ,and cumulative incidence function of castration-resistant prostate cancer (C) of the H pylori infection patients with early or late-eradication in the propensity score matched cohort.

**Supplementary Table 1**. The ICD-9 and ICD-10 diagnostic codes

| **Variable** | **ICD-9-CM** | **ICD-10-CM** |
| --- | --- | --- |
| **Select study cohort** |  |  |
| Prostate cancer | 185.xx or ICD-O-3: C619 with Histology M code: 814, 820, 821, 825, 826, 831, 848, 850, 851, 855, 856, 857 | C61.xx or ICD-O-3: C619 with Histology M code: 814, 820, 821, 825, 826, 831, 848, 850, 851, 855, 856, 857 |
| *H. pylori* infection | 531.xx-533.xx with eradication therapy | K25-K27, K31.82 with eradication therapy |
| **Peptic ulcer site** |  |  |
| Gastric ulcer | 531.xx | K25, K31.82 |
| Duodenal ulcer | 532.xx | K26, K31.82 |
| Other site | 533.xx | K27 |
| **Complicated peptic ulcer** |  |  |
| Gastric ulcer bleeding | 531.xx except 531.3, 531.7 | K31.82, K25 except K25.3, K25.7, K25.9 |
| Duodenal ulcer bleeding | 532.xx except 532.3, 532.7 | K31.82, K26 except K26.3, K26.7, K26.9 |
| Other site bleeding | 533.xx except 533.3, 533.7 | K27 except K27.3, K27.7, K27.9 |
| **Comorbidities** |  |  |
| Hypertension | 401.xx-405.xx with any anti-hypertension drugs | I10-I15, N262 with any anti-hypertension drugs |
| Diabetes | 250.xx with any oral hypoglycemic drugs | E08-E13 with any oral hypoglycemic drugs and insulin |
| Coronary heart disease | 410.xx-414.xx | I20-I24 |
| Old myocardial infarction | 410.xx, 412.xx | I21-I22 |
| Heart failure | 428.xx | I50 |
| Hyperlipidemia | 272.0-272.4 with any anti-hyperlipidemia drugs | E77, E780, E781, E782, E783, E784, E785, E786, E881, E753, E755, E882, E756, E789, E7521, E7522, E7524, E7130, E7879, E7881, E7889, E8889, E7870 with any anti-hyperlipidemia drugs |
| Atrial fibrillation | 427.3x | I48 |
| Peripheral arterial disease | 440.xx, 441.xx, 443.xx, 444.0x, 444.8x, 447.8x, 447.9x, 093.0, 437.3, 444.22, 447.1, 557.1, 557.9, V434 | I70, I71, I73, I75, I771, I790, I791, I792, I773, I779, I798, K551, K558, K559, Z958, Z959, I743, I744, I745, I748, I740, I7789 |
| Cerebrovascular disease | 430.xx-437.xx | I60-I69 |
| Chronic obstructive pulmonary disease | 491.xx, 492.xx, 496.xx | J41-J44 |
| Chronic kidney disease | 580.xx-589.xx, 403.xx-404.xx, 016.0x, 095.4x, 236.9x, 250.4x, 274.1x, 442.1x, 447.3x, 440.1x, 572.4x, 642.1x, 646.2x, 753.1x, 283.11, 403.01, 404.02, 446.21 | A1811, D593, E102, E112, E132, I12, I13, K767, M103, M310, N00, N01, N02, N03, N04, N05, N06, N07, N08, N14, N150, N158, N159, N16, N171, N172, N18, N19, N200, N25, N261, N269, N27, Q61 |
| Chronic liver disease | 070.xx, 456.0-456.2, 570.xx, 571.xx,  572.2-572.8, 573.xx, V42.7 | B15, B16, B17, B18, B19, I85, K70, K71, K72, K73, K74, K752, K753, K754, K758, K759, K76, K77, Z944 |
| Alcoholism | 571.2 | K70, F1010, F1012, F1014, F1015, F1018, F1019, F1020, F1021, F1022, F1023, F1024, F1025, F1026, F1027, F1028, F1029, F1092, F1094, F1095, F1096, F1097, F1098, F1099, G621, I426, T510X1A, T510X2A, T510X3A, T510X4A, Z658 |

ICD-9-CM, International Classification of Diseases, Ninth Revision, Clinical Modification; ICD-O-3, International Classification of Diseases for Oncology, Third Edition; ICD-10-CM, International Classification of Diseases, Tenth Revision, Clinical Modification.

**Supplementary Table 2**. *Helicobacter pylori* eradication regimens

| Type Helicobacter pylori Eradication Regimen |
| --- |
| 1. PPI + clarithromycin + amoxicillin |
| 1. PPI + clarithromycin + metronidazole |
| 1. PPI + amoxicillin + metronidazole |
| 1. PPI + tetracyclin + amoxicillin |
| 1. PPI + tetracyclin + metronidazole |
| 1. PPI + clarithromycin + tetracycline |
| 1. PPI + bismuth + tetracyclin + metronidazole |
| 1. PPI + bismuth + amoxicillin + metronidazole |
| 1. PPI + levofloxacin + amoxicillin |
| 1. PPI + bismuth + tetracyclin + levofloxacin |
| 1. PPI + bismuth + amoxicillin + levofloxacin |
| 1. H2RA + clarithromycin + amoxicillin |
| 1. H2RA + clarithromycin + metronidazole |
| 1. H2RA + amoxicillin + metronidazole |
| 1. H2RA + tetracyclin + amoxicillin |
| 1. H2RA + tetracyclin + metronidazole |
| 1. H2RA + clarithromycin + tetracycline |
| 1. H2RA + bismuth + tetracyclin + metronidazole |
| 1. H2RA + bismuth + amoxicillin + metronidazole |
| 1. H2RA + levofloxacin + amoxicillin |
| 1. H2RA + bismuth + tetracyclin + levofloxacin |
| 1. H2RA + bismuth + amoxicillin + levofloxacin |
| 1. PPI + clarithromycin + bismuth |
| 1. PPI + amoxicillin + bismuth |
| 1. PPI + metronidazole + bismuth |
| 1. PPI + tetracyclin + bismuth |
| 1. H2RA + clarithromycin + bismuth |
| 1. H2RA + amoxicillin + bismuth |
| 1. H2RA + metronidazole + bismuth |
| 1. H2RA + tetracyclin + bismuth |

These drug combinations should be prescribed within the same prescription order and the duration of therapy was between 7 and 14 days;

PPI, proton pump inhibitor; H2RA, H2 receptor antagonist.

**Supplementary Table 3**. The ATC codes for pharmacotherapy used in this study

| Medication | ATC code |
| --- | --- |
| Gonadotropin releasing hormones (GnRH agonists) | L02AE, H01CA |
| Oral antiandrogens | L02BB01, L02BB02, L02BB03 |
| Docetaxel | L01CD02 |
| Enzalutamide | L02BB04 |
| Abiraterone acetate | L02BX03 |
| Proton pump inhibitor | A02BC |
| H2-receptor antagonists | A02BA |
| Clarithromycin | J01FA09 |
| Amoxicillin | J01CA04 |
| Metronidazole | J01XD01, P01AB01 |
| Tetracyclines | J01AA07 |
| Bismuth subcitrate | A02BX05 |
| Levofloxacin | J01MA12 |
| Combinations for eradication of Helicobacter pylori | A02BD |
| Non-steroidal anti-inflammatory drug | M01AA, M01AB, M01AC, M01AE, M01AG, M01AX |
| Aspirin | A01AD05, B01AC06, N02BA01 |
| Clopidogrel | B01AC04 |
| Cox-2 inhibitor | M01AH |
| Anticoagulant agents | B01AA |
| Oral hypoglycemic agent | A10BA, A10BB, A10BG02, A10BG03, A10BH, A10BF |
| Insulin | A10A |
| ACEI / ARB | C09 |

ACT, Anatomical Therapeutic Chemical code; ACEi/ARB, angiotensin-converting-enzyme inhibitor / angiotensin receptor blockers.

**Supplementary Table 4**. Characteristics of the patients with and without *H. pylori* infection before and after inverse probability treatment weight

|  | Before IPTW‡ | | |  | After IPTW† | | |
| --- | --- | --- | --- | --- | --- | --- | --- |
| Variables | *H. pylori*  (*n* = 3516) | Non-*H. pylori*  (*n* = 20185) | STD |  | *H. pylori* | Non-*H. pylori* | STD |
| Age (year) | 74.4±8.2 | 74.0±8.4 | 0.056 |  | 73.8±20.7 | 74.0±9.1 | -0.013 |
| Age group |  |  |  |  |  |  |  |
| 40-49 years | 12 (0.3) | 104 (0.5) | -0.028 |  | 0.4% | 0.5% | -0.010 |
| 50-59 years | 165 (4.7) | 1180 (5.9) | -0.052 |  | 5.6% | 5.7% | -0.003 |
| 60-69 years | 799 (22.7) | 4698 (23.3) | -0.013 |  | 25.7% | 23.2% | 0.057 |
| 70-79 years | 1605 (45.7) | 9295 (46.1) | -0.008 |  | 44.2% | 46.0% | -0.035 |
| ≥80 years | 935 (26.6) | 4908 (24.3) | 0.052 |  | 24.0% | 24.6% | -0.014 |
| Urbanization |  |  |  |  |  |  |  |
| 1 (most urbanized) | 757 (21.5) | 4647 (23.0) | -0.036 |  | 21.0% | 22.8% | -0.044 |
| 2 | 1387 (39.5) | 7717 (38.2) | 0.025 |  | 37.6% | 38.4% | -0.016 |
| 3 | 902 (25.7) | 5054 (25.0) | 0.014 |  | 28.0% | 25.2% | 0.064 |
| 4 (least urbanized) | 470 (13.4) | 2767 (13.7) | -0.010 |  | 13.4% | 13.6% | -0.006 |
| Monthly income, NT$ |  |  |  |  |  |  |  |
| Dependent | 5 (0.1) | 22 (0.1) | 0.008 |  | 0.1% | 0.1% | 0.006 |
| < 15,000 | 1304 (37.1) | 7737 (38.3) | -0.026 |  | 35.8% | 38.1% | -0.049 |
| 15,000-24,999 | 1350 (38.4) | 7814 (38.7) | -0.006 |  | 41.7% | 38.7% | 0.061 |
| ≥25,000 | 857 (24.4) | 4612 (22.9) | 0.036 |  | 22.4% | 23.1% | -0.016 |
| Comorbidity |  |  |  |  |  |  |  |
| Hypertension | 2102 (59.8) | 8541 (42.3) | 0.355 |  | 50.6% | 44.9% | 0.115 |
| Diabetes | 813 (23.1) | 3160 (15.7) | 0.190 |  | 18.8% | 16.8% | 0.054 |
| Coronary heart disease | 795 (22.6) | 2855 (14.1) | 0.220 |  | 15.9% | 15.4% | 0.014 |
| Hyperlipidemia | 536 (15.2) | 1687 (8.4) | 0.214 |  | 10.2% | 9.4% | 0.029 |
| Atrial fibrillation | 115 (3.3) | 487 (2.4) | 0.052 |  | 3.0% | 2.6% | 0.025 |
| Peripheral arterial disease | 80 (2.3) | 273 (1.4) | 0.070 |  | 1.4% | 1.5% | -0.004 |
| COPD | 518 (14.7) | 2007 (9.9) | 0.146 |  | 12.4% | 10.7% | 0.055 |
| Chronic kidney disease | 477 (13.6) | 1729 (8.6) | 0.160 |  | 10.2% | 9.3% | 0.028 |
| Chronic liver disease | 453 (12.9) | 1146 (5.7) | 0.250 |  | 8.8% | 6.8% | 0.073 |
| Alcoholism | 17 (0.5) | 60 (0.3) | 0.029 |  | 0.5% | 0.3% | 0.019 |
| History of event |  |  |  |  |  |  |  |
| Old myocardial infarction | 101 (2.9) | 336 (1.7) | 0.081 |  | 2.2% | 1.9% | 0.025 |
| Heart failure | 151 (4.3) | 513 (2.5) | 0.096 |  | 3.5% | 2.8% | 0.039 |
| Cerebrovascular disease | 351 (10.0) | 1264 (6.3) | 0.137 |  | 7.5% | 6.8% | 0.026 |
| CCI score | 3.9±2.5 | 2.9±2.5 | 0.399 |  | 3.5±6.3 | 3.0±2.7 | 0.088 |
| CCI score group |  |  |  |  |  |  |  |
| 0 | 95 (2.7) | 3125 (15.5) | -0.456 |  | 5.0% | 13.6% | -0.300 |
| 1-2 | 955 (27.2) | 8352 (41.4) | -0.303 |  | 38.9% | 39.2% | -0.007 |
| ≥3 | 2466 (70.1) | 8708 (43.1) | 0.566 |  | 56.2% | 47.2% | 0.180 |
| Prostate cancer stage at diagnosis |  |  |  |  |  |  |  |
| I | 107 (3.0) | 239 (1.2) | 0.130 |  | 1.6% | 1.5% | 0.012 |
| II | 801 (22.8) | 2376 (11.8) | 0.294 |  | 15.3% | 13.4% | 0.054 |
| III | 417 (11.9) | 1358 (6.7) | 0.177 |  | 8.7% | 7.5% | 0.042 |
| IV | 737 (21.0) | 2932 (14.5) | 0.169 |  | 17.5% | 15.5% | 0.054 |
| Unknown (data before 2007) | 1454 (41.4) | 13280 (65.8) | -0.505 |  | 56.9% | 62.1% | -0.106 |
| Disease extent |  |  |  |  |  |  |  |
| Loco regional | 2617 (74.4) | 16066 (79.6) | -0.123 |  | 77.2% | 78.8% | -0.039 |
| Distant metastasis | 899 (25.6) | 4119 (20.4) | 0.123 |  | 22.8% | 21.2% | 0.039 |
| Medication |  |  |  |  |  |  |  |
| NSAID | 401 (11.4) | 1424 (7.1) | 0.151 |  | 8.2% | 7.7% | 0.018 |
| Aspirin | 885 (25.2) | 4735 (23.5) | 0.040 |  | 25.0% | 23.8% | 0.028 |
| Clopidogrel | 272 (7.7) | 351 (1.7) | 0.285 |  | 2.8% | 2.6% | 0.014 |
| Cox-2 inhibitor | 219 (6.2) | 450 (2.2) | 0.200 |  | 3.1% | 2.8% | 0.019 |
| Anticoagulant agents | 70 (2.0) | 322 (1.6) | 0.029 |  | 1.6% | 1.7% | -0.006 |
| Oral hypoglycemic agent | 1058 (30.1) | 4623 (22.9) | 0.163 |  | 25.9% | 24.0% | 0.045 |
| Insulin | 101 (2.9) | 402 (2.0) | 0.057 |  | 2.1% | 2.1% | -0.001 |
| ACEI / ARB | 1408 (40.1) | 5611 (27.8) | 0.261 |  | 31.0% | 29.6% | 0.032 |
| Follow-up years | 4.6±3.1 | 5.7±4.2 | -0.298 |  | 5.5±9.3 | 5.6±4.5 | -0.002 |

*H.pylori, Helicobacter pylori* ;STD, standardized difference; COPD, chronic obstructive pulmonary disease CCI, Charlson Comorbidity Index; ADT, androgen deprivation therapy; NSAID, non-steroidal anti-inflammatory drug; ACEI, angiotensin converting enzyme inhibitor; ARB, angiotensin II receptor blockers; GnRH, gonadotropin releasing hormone;

‡ Value are given as number (%) or mean±SD;

† Values are given as % or mean±SD.

**Supplementary Table 5**. Characteristics of the *H. pylori* infection patients with early or late eradication before and after propensity score matching

|  | Before matching | | |  | After matching | | |
| --- | --- | --- | --- | --- | --- | --- | --- |
| Variables | Early-eradication  (*n* = 2062) | Late-eradication  (*n* = 1454) | STD |  | Early-eradication  (*n* = 1192) | Late-eradication  (*n* = 1192) | STD |
| Age (year) | 73.8±8.2 | 75.4±8.1 | -0.199 |  | 74.8±8.2 | 74.8±8.1 | -0.008 |
| Age group |  |  |  |  |  |  |  |
| 40-49 years | 9 (0.4) | 3 (0.2) | 0.040 |  | 6 (0.5) | 3 (0.3) | 0.041 |
| 50-59 years | 112 (5.4) | 53 (3.7) | 0.086 |  | 46 (3.9) | 49 (4.1) | -0.013 |
| 60-69 years | 509 (24.7) | 290 (19.9) | 0.114 |  | 264 (22.2) | 257 (21.6) | 0.014 |
| 70-79 years | 950 (46.1) | 655 (45.1) | 0.020 |  | 543 (45.6) | 552 (46.3) | -0.015 |
| ≥80 years | 482 (23.4) | 453 (31.2) | -0.175 |  | 333 (27.9) | 331 (27.8) | 0.004 |
| Urbanization |  |  |  |  |  |  |  |
| 1 (most urbanized) | 443 (21.5) | 314 (21.6) | -0.003 |  | 263 (22.1) | 258 (21.6) | 0.010 |
| 2 | 826 (40.1) | 561 (38.6) | 0.030 |  | 467 (39.2) | 466 (39.1) | 0.002 |
| 3 | 535 (26.0) | 367 (25.2) | 0.016 |  | 298 (25.0) | 306 (25.7) | -0.015 |
| 4 (least urbanized) | 258 (12.5) | 212 (14.6) | -0.061 |  | 164 (13.8) | 162 (13.6) | 0.005 |
| Monthly income, NT$ |  |  |  |  |  |  |  |
| < 15,000 or dependent | 718 (34.8) | 591 (40.6) | -0.120 |  | 459 (38.5) | 457 (38.3) | 0.003 |
| 15,000-24,999 | 805 (39.0) | 545 (37.5) | 0.032 |  | 457 (38.3) | 460 (38.6) | -0.005 |
| ≥25,000 | 539 (26.1) | 318 (21.9) | 0.100 |  | 276 (23.2) | 275 (23.1) | 0.002 |
| CCI score | 3.8±2.4 | 4.1±2.5 | -0.134 |  | 3.8±2.4 | 4.0±2.6 | -0.070 |
| CCI score group |  |  |  |  |  |  |  |
| 0 | 61 (3.0) | 34 (2.3) | 0.039 |  | 32 (2.7) | 30 (2.5) | 0.010 |
| 1-2 | 614 (29.8) | 341 (23.5) | 0.144 |  | 326 (27.4) | 314 (26.3) | 0.023 |
| ≥3 | 1387 (67.3) | 1079 (74.2) | -0.153 |  | 834 (70.0) | 848 (71.1) | -0.026 |
| Peptic ulcer sites |  |  |  |  |  |  |  |
| Gastric ulcer | 1220 (59.2) | 1083 (74.5) | -0.330 |  | 815 (68.4) | 834 (70.0) | -0.035 |
| Duodenal ulcer | 1046 (50.7) | 872 (60.0) | -0.187 |  | 666 (55.9) | 662 (55.5) | 0.007 |
| Other site | 1628 (79.0) | 1384 (95.2) | -0.499 |  | 1128 (94.6) | 1122 (94.1) | 0.022 |
| Comorbidity |  |  |  |  |  |  |  |
| Hypertension | 1171 (56.8) | 931 (64.0) | -0.148 |  | 723 (60.7) | 730 (61.2) | -0.012 |
| Diabetes | 480 (23.3) | 333 (22.9) | 0.009 |  | 283 (23.7) | 276 (23.2) | 0.014 |
| Coronary heart disease | 438 (21.2) | 357 (24.6) | -0.079 |  | 278 (23.3) | 291 (24.4) | -0.026 |
| Hyperlipidemia | 323 (15.7) | 213 (14.7) | 0.028 |  | 180 (15.1) | 184 (15.4) | -0.009 |
| Atrial fibrillation | 62 (3.0) | 53 (3.7) | -0.036 |  | 38 (3.2) | 40 (3.4) | -0.010 |
| Peripheral arterial disease | 48 (2.3) | 32 (2.2) | 0.009 |  | 26 (2.2) | 27 (2.3) | -0.006 |
| COPD | 269 (13.1) | 249 (17.1) | -0.114 |  | 180 (15.1) | 183 (15.4) | -0.007 |
| Chronic kidney disease | 251 (12.2) | 226 (15.5) | -0.098 |  | 163 (13.7) | 178 (14.9) | -0.036 |
| Chronic liver disease | 231 (11.2) | 222 (15.3) | -0.120 |  | 161 (13.5) | 166 (13.9) | -0.012 |
| Alcoholism | 11 (0.5) | 6 (0.4) | 0.018 |  | 4 (0.3) | 6 (0.5) | -0.025 |
| History of event |  |  |  |  |  |  |  |
| Old myocardial infarction | 53 (2.6) | 48 (3.3) | -0.043 |  | 35 (2.9) | 35 (2.9) | 0.000 |
| Heart failure | 77 (3.7) | 74 (5.1) | -0.066 |  | 56 (4.7) | 56 (4.7) | 0.000 |
| Cerebrovascular disease | 194 (9.4) | 157 (10.8) | -0.046 |  | 127 (10.7) | 132 (11.1) | -0.013 |
| CCI score | 3.8±2.4 | 4.1±2.5 | -0.134 |  | 3.8±2.4 | 4.0±2.6 | -0.070 |
| CCI score group |  |  |  |  |  |  |  |
| 0 | 61 (3.0) | 34 (2.3) | 0.039 |  | 32 (2.7) | 30 (2.5) | 0.010 |
| 1-2 | 614 (29.8) | 341 (23.5) | 0.144 |  | 326 (27.4) | 314 (26.3) | 0.023 |
| ≥3 | 1387 (67.3) | 1079 (74.2) | -0.153 |  | 834 (70.0) | 848 (71.1) | -0.026 |
| Prostate cancer stage at diagnosis |  |  |  |  |  |  |  |
| I | 48 (2.3) | 59 (4.1) | -0.098 |  | 38 (3.2) | 38 (3.2) | 0.000 |
| II | 464 (22.5) | 337 (23.2) | -0.016 |  | 287 (24.1) | 283 (23.7) | 0.008 |
| III | 228 (11.1) | 189 (13.0) | -0.060 |  | 150 (12.6) | 148 (12.4) | 0.005 |
| IV | 432 (21.0) | 305 (21.0) | -0.001 |  | 257 (21.6) | 256 (21.5) | 0.002 |
| Unknown (data before 2007) | 890 (43.2) | 564 (38.8) | 0.089 |  | 460 (38.6) | 467 (39.2) | -0.012 |
| Disease extent |  |  |  |  |  |  |  |
| Loco regional | 1530 (74.2) | 1087 (74.8) | -0.013 |  | 886 (74.3) | 882 (74.0) | 0.008 |
| Distant metastasis | 532 (25.8) | 367 (25.2) | 0.013 |  | 306 (25.7) | 310 (26.0) | -0.008 |
| Medication |  |  |  |  |  |  |  |
| NSAID | 203 (9.8) | 198 (13.6) | -0.118 |  | 146 (12.3) | 146 (12.3) | 0.000 |
| Aspirin | 511 (24.8) | 374 (25.7) | -0.022 |  | 301 (25.3) | 317 (26.6) | -0.031 |
| Clopidogrel | 145 (7.0) | 127 (8.7) | -0.063 |  | 100 (8.4) | 101 (8.5) | -0.003 |
| Cox-2 inhibitor | 111 (5.4) | 108 (7.4) | -0.084 |  | 79 (6.6) | 85 (7.1) | -0.020 |
| Anticoagulant agents | 36 (1.8) | 34 (2.3) | -0.042 |  | 24 (2.0) | 23 (1.9) | 0.006 |
| OHA | 620 (30.1) | 438 (30.1) | -0.001 |  | 368 (30.9) | 361 (30.3) | 0.013 |
| Insulin | 60 (2.9) | 41 (2.8) | 0.005 |  | 29 (2.4) | 35 (2.9) | -0.032 |
| ACEI / ARB | 776 (37.6) | 632 (43.5) | -0.119 |  | 487 (40.9) | 498 (41.8) | -0.019 |
| Follow-up years | 4.8±3.2 | 4.4±2.9 | 0.132 |  | 4.5±2.9 | 4.6±2.9 | -0.040 |

*H.pylori, Helicobacter pylori* ; STD, standardized difference; COPD, chronic obstructive pulmonary disease CCI, Charlson Comorbidity Index; ADT, androgen deprivation therapy; NSAID, non-steroidal anti-inflammatory drug; OHA, oral hypoglycemic agent; ACEI, angiotensin converting enzyme inhibitor; ARB, angiotensin II receptor blockers; GnRH, gonadotropin releasing hormone; NA, not applicable.

**Supplementary Table 6**. Characteristics of the *H. pylori* infection patients with early or late-eradication before and after inverse probability treatment weight

|  | Before IPTW‡ | | |  | After IPTW† | | |
| --- | --- | --- | --- | --- | --- | --- | --- |
| Variables | Early-eradication  (*n* = 2062) | Late-eradication  (*n* = 1454) | STD |  | Early-eradication | Late-eradication | STD |
| Age (year) | 73.8±8.2 | 75.4±8.1 | -0.199 |  | 74.4±10.7 | 74.2±12.8 | 0.011 |
| Age group |  |  |  |  |  |  |  |
| 40-49 | 9 (0.4) | 3 (0.2) | 0.040 |  | 0.3% | 0.3% | 0.013 |
| 50-59 | 112 (5.4) | 53 (3.7) | 0.086 |  | 4.7% | 5.0% | -0.015 |
| 60-69 | 509 (24.7) | 290 (19.9) | 0.114 |  | 23.2% | 24.2% | -0.023 |
| 70-79 | 950 (46.1) | 655 (45.1) | 0.020 |  | 45.5% | 44.6% | 0.020 |
| ≥80 | 482 (23.4) | 453 (31.2) | -0.175 |  | 26.3% | 26.0% | 0.006 |
| Urbanization |  |  |  |  |  |  |  |
| 1 (most urbanized) | 443 (21.5) | 314 (21.6) | -0.003 |  | 21.5% | 21.7% | -0.004 |
| 2 | 826 (40.1) | 561 (38.6) | 0.030 |  | 39.4% | 38.4% | 0.022 |
| 3 | 535 (26.0) | 367 (25.2) | 0.016 |  | 25.7% | 26.6% | -0.019 |
| 4 (least urbanized) | 258 (12.5) | 212 (14.6) | -0.061 |  | 13.3% | 13.4% | -0.002 |
| Monthly income, NT$ |  |  |  |  |  |  |  |
| < 15,000 or dependent | 718 (34.8) | 591 (40.6) | -0.120 |  | 36.8% | 36.9% | -0.002 |
| 15,000-24,999 | 805 (39.0) | 545 (37.5) | 0.032 |  | 38.6% | 38.2% | 0.008 |
| ≥25,000 | 539 (26.1) | 318 (21.9) | 0.100 |  | 24.6% | 24.9% | -0.007 |
| Peptic ulcer sites |  |  |  |  |  |  |  |
| Gastric ulcer | 1220 (59.2) | 1083 (74.5) | -0.330 |  | 65.5% | 66.7% | -0.026 |
| Duodenal ulcer | 1046 (50.7) | 872 (60.0) | -0.187 |  | 54.9% | 54.3% | 0.012 |
| Other site | 1628 (79.0) | 1384 (95.2) | -0.499 |  | 85.7% | 86.0% | -0.011 |
| Comorbidity |  |  |  |  |  |  |  |
| Hypertension | 1171 (56.8) | 931 (64.0) | -0.148 |  | 59.6% | 60.2% | -0.012 |
| Diabetes | 480 (23.3) | 333 (22.9) | 0.009 |  | 23.1% | 23.3% | -0.005 |
| Coronary heart disease | 438 (21.2) | 357 (24.6) | -0.079 |  | 22.5% | 23.7% | -0.029 |
| Hyperlipidemia | 323 (15.7) | 213 (14.7) | 0.028 |  | 15.3% | 16.1% | -0.021 |
| Atrial fibrillation | 62 (3.0) | 53 (3.7) | -0.036 |  | 3.2% | 3.5% | -0.017 |
| Peripheral arterial disease | 48 (2.3) | 32 (2.2) | 0.009 |  | 2.3% | 2.3% | -0.002 |
| COPD | 269 (13.1) | 249 (17.1) | -0.114 |  | 14.8% | 14.8% | -0.002 |
| Chronic kidney disease | 251 (12.2) | 226 (15.5) | -0.098 |  | 13.7% | 13.5% | 0.004 |
| Chronic liver disease | 231 (11.2) | 222 (15.3) | -0.120 |  | 13.0% | 12.9% | 0.002 |
| Alcoholism | 11 (0.5) | 6 (0.4) | 0.018 |  | 0.5% | 0.4% | 0.011 |
| History of event |  |  |  |  |  |  |  |
| Old myocardial infarction | 53 (2.6) | 48 (3.3) | -0.043 |  | 2.7% | 2.6% | 0.007 |
| Heart failure | 77 (3.7) | 74 (5.1) | -0.066 |  | 4.1% | 4.0% | 0.006 |
| Cerebrovascular disease | 194 (9.4) | 157 (10.8) | -0.046 |  | 10.0% | 10.9% | -0.028 |
| CCI score | 3.8±2.4 | 4.1±2.5 | -0.134 |  | 3.8±3.2 | 4.0±4.1 | -0.034 |
| CCI score group |  |  |  |  |  |  |  |
| 0 | 61 (3.0) | 34 (2.3) | 0.039 |  | 2.7% | 2.8% | -0.003 |
| 1-2 | 614 (29.8) | 341 (23.5) | 0.144 |  | 27.1% | 27.5% | -0.007 |
| ≥3 | 1387 (67.3) | 1079 (74.2) | -0.153 |  | 70.1% | 69.8% | 0.009 |
| Prostate cancer stage at diagnosis |  |  |  |  |  |  |  |
| I | 48 (2.3) | 59 (4.1) | -0.098 |  | 3.0% | 3.1% | -0.006 |
| II | 464 (22.5) | 337 (23.2) | -0.016 |  | 22.9% | 23.0% | -0.003 |
| III | 228 (11.1) | 189 (13.0) | -0.060 |  | 11.8% | 11.5% | 0.010 |
| IV | 432 (21.0) | 305 (21.0) | -0.001 |  | 20.9% | 20.6% | 0.008 |
| Unknown | 890 (43.2) | 564 (38.8) | 0.089 |  | 41.4% | 41.9% | -0.008 |
| Disease extent |  |  |  |  |  |  |  |
| Loco regional | 1530 (74.2) | 1087 (74.8) | -0.013 |  | 74.6% | 74.7% | -0.004 |
| Distant metastasis | 532 (25.8) | 367 (25.2) | 0.013 |  | 25.5% | 25.3% | 0.004 |
| Medication |  |  |  |  |  |  |  |
| NSAID | 203 (9.8) | 198 (13.6) | -0.118 |  | 11.4% | 12.0% | -0.020 |
| Aspirin | 511 (24.8) | 374 (25.7) | -0.022 |  | 25.3% | 26.3% | -0.024 |
| Clopidogrel | 145 (7.0) | 127 (8.7) | -0.063 |  | 7.6% | 7.7% | -0.004 |
| Cox-2 inhibitor | 111 (5.4) | 108 (7.4) | -0.084 |  | 6.1% | 5.9% | 0.011 |
| Anticoagulant agents | 36 (1.8) | 34 (2.3) | -0.042 |  | 2.0% | 2.2% | -0.013 |
| OHA | 620 (30.1) | 438 (30.1) | -0.001 |  | 30.0% | 30.2% | -0.003 |
| Insulin | 60 (2.9) | 41 (2.8) | 0.005 |  | 2.8% | 2.5% | 0.015 |
| ACEI / ARB | 776 (37.6) | 632 (43.5) | -0.119 |  | 40.2% | 41.2% | -0.021 |
| Propensity score | 0.644±0.179 | 0.505±0.157 | 0.822 |  | 0.587±0.238 | 0.583±0.279 | 0.015 |
| Follow-up years | 4.8±3.2 | 4.4±2.9 | 0.132 |  | 4.6±4.0 | 4.8±4.8 | -0.029 |

*H.pylori, Helicobacter pylori* ; STD, standardized difference; COPD, chronic obstructive pulmonary disease CCI, Charlson Comorbidity Index; ADT, androgen deprivation therapy; NSAID, non-steroidal anti-inflammatory drug; OHA, oral hypoglycemic agent; ACEI, angiotensin converting enzyme inhibitor; ARB, angiotensin II receptor blockers; GnRH, gonadotropin releasing hormone; NA, not applicable;

‡ Value are given as number (%) or mean±SD;

† Values are given as % or mean±SD.

**Supplementary Table 7**. Follow-up outcomes of the *H. pylori* infection patients with early or late-eradication

|  | Early-eradication | |  | Late-eradication | |  | HR of early (95% CI) |
| --- | --- | --- | --- | --- | --- | --- | --- |
| Outcomes | Event (%) | Incidence density |  | Event (%) | Incidence density |  |  |
| Primary analysis: PSM |  |  |  |  |  |  |  |
| All-cause mortality | 483 (40.5) | 9.09 (8.28–9.91) |  | 467 (39.2) | 8.57 (7.79–9.34) |  | 1.06 (0.94, 1.21) |
| Prostate cancer-specific mortality | 332 (27.9) | 6.25 (5.58–6.92) |  | 331 (27.8) | 6.07 (5.42–6.73) |  | 1.03 (0.88, 1.20) |
| CRPC# | 76 (6.4) | 1.45 (1.13–1.78) |  | 79 (6.6) | 1.48 (1.15–1.80) |  | 0.97 (0.71, 1.33) |
| Sensitivity analysis: IPTW† |  |  |  |  |  |  |  |
| All-cause mortality | 41.5% | 8.98 (8.52–9.45) |  | 39.7% | 8.36 (7.92–8.80) |  | 1.08 (0.95, 1.22) |
| Prostate cancer-specific mortality | 29.0% | 6.27 (5.88–6.65) |  | 27.9% | 5.86 (5.49–6.23) |  | 1.07 (0.93, 1.24) |
| CRPC# | 7.3% | 1.60 (1.41–1.80) |  | 7.9% | 1.71 (1.51–1.91) |  | 0.92 (0.67, 1.28) |

*H.pylori, Helicobacter pylori* ; HR, hazard ratio; CI, confidence interval; PSM, propensity score matching; IPTW, inverse-probability-of-treatment weighting; CRPC, castration-resistant prostate cancer;

† Event values are given as %;

# Estimated using the subdistribution hazard model which considered all-cause death as a competing risk.


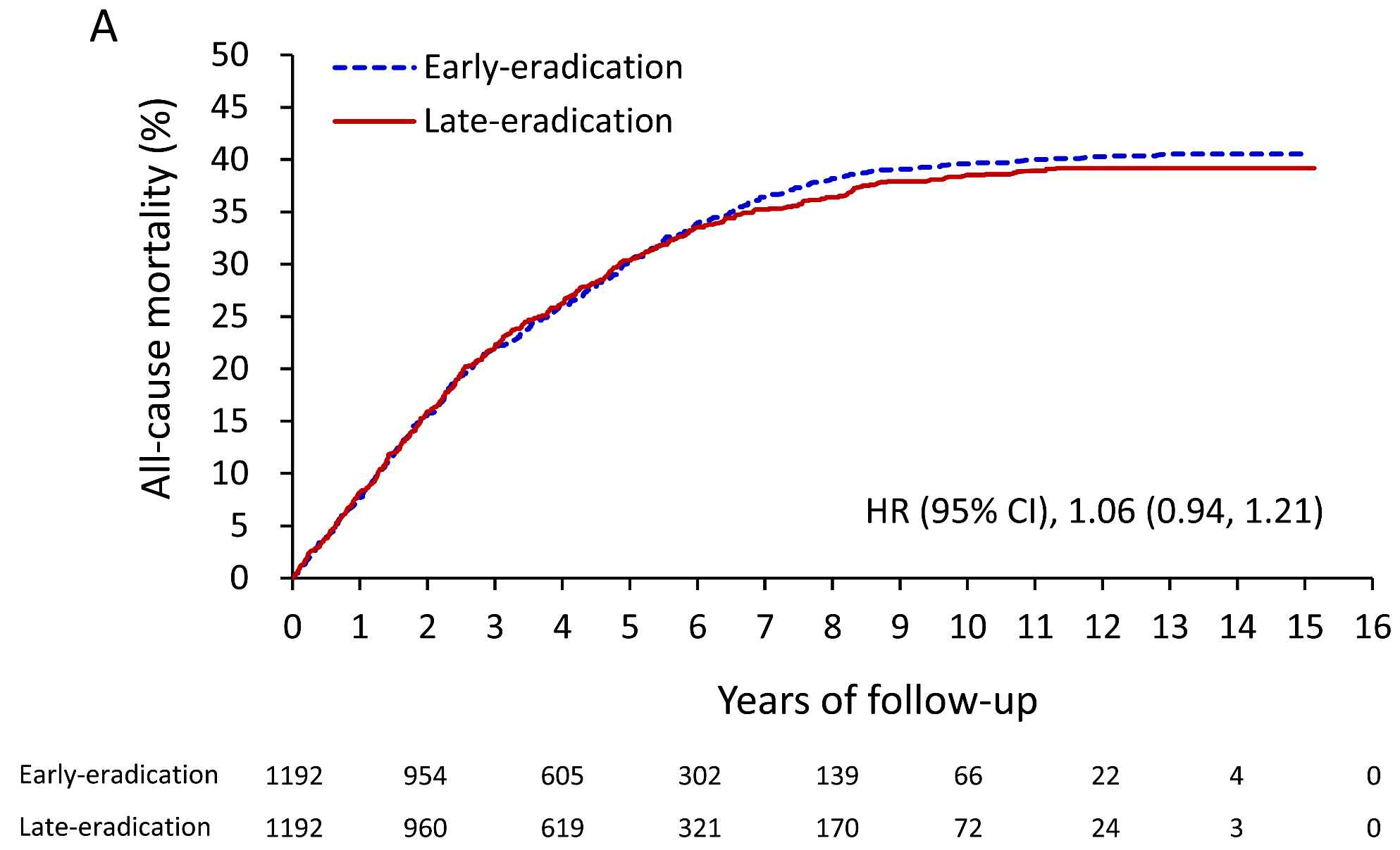


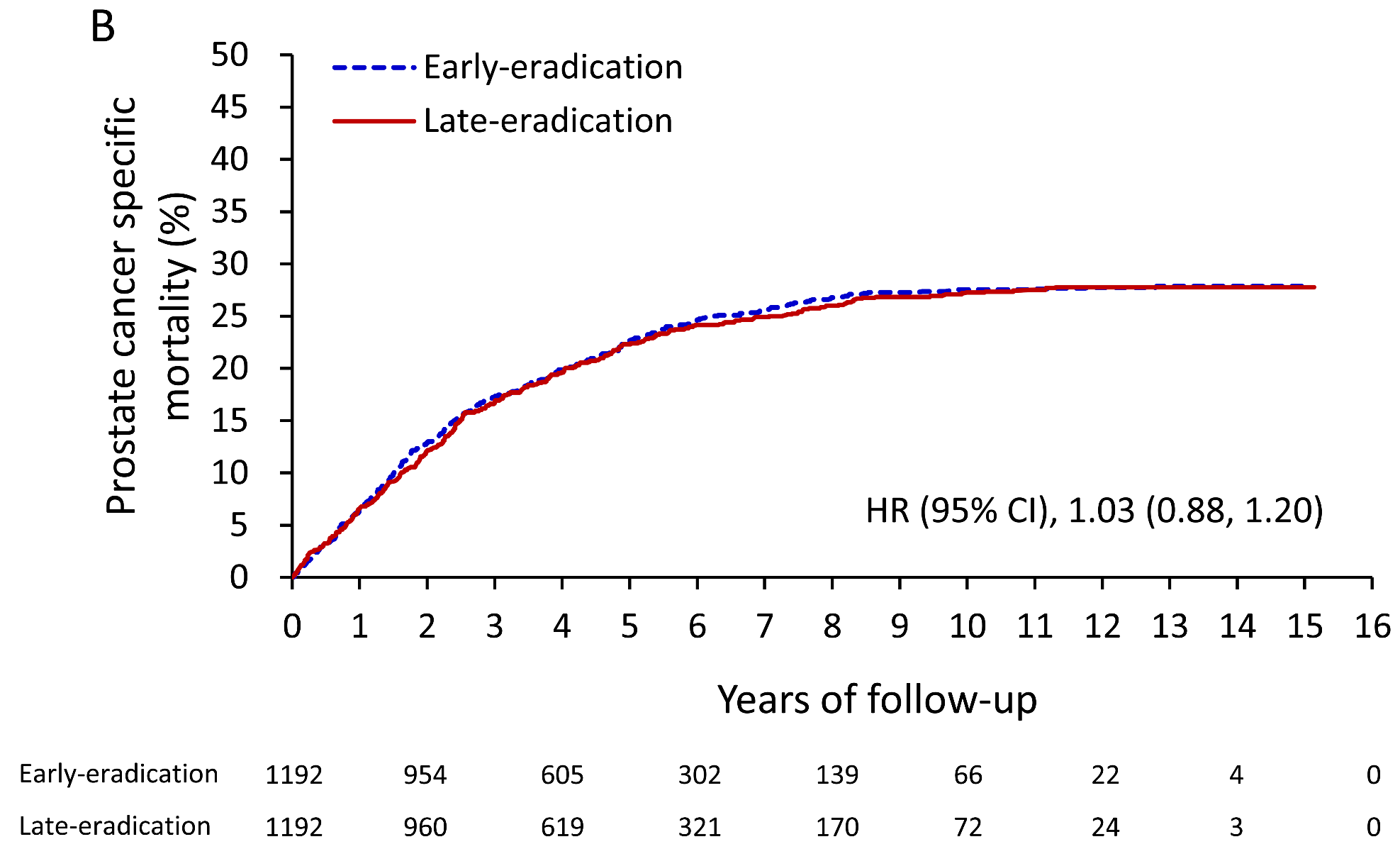


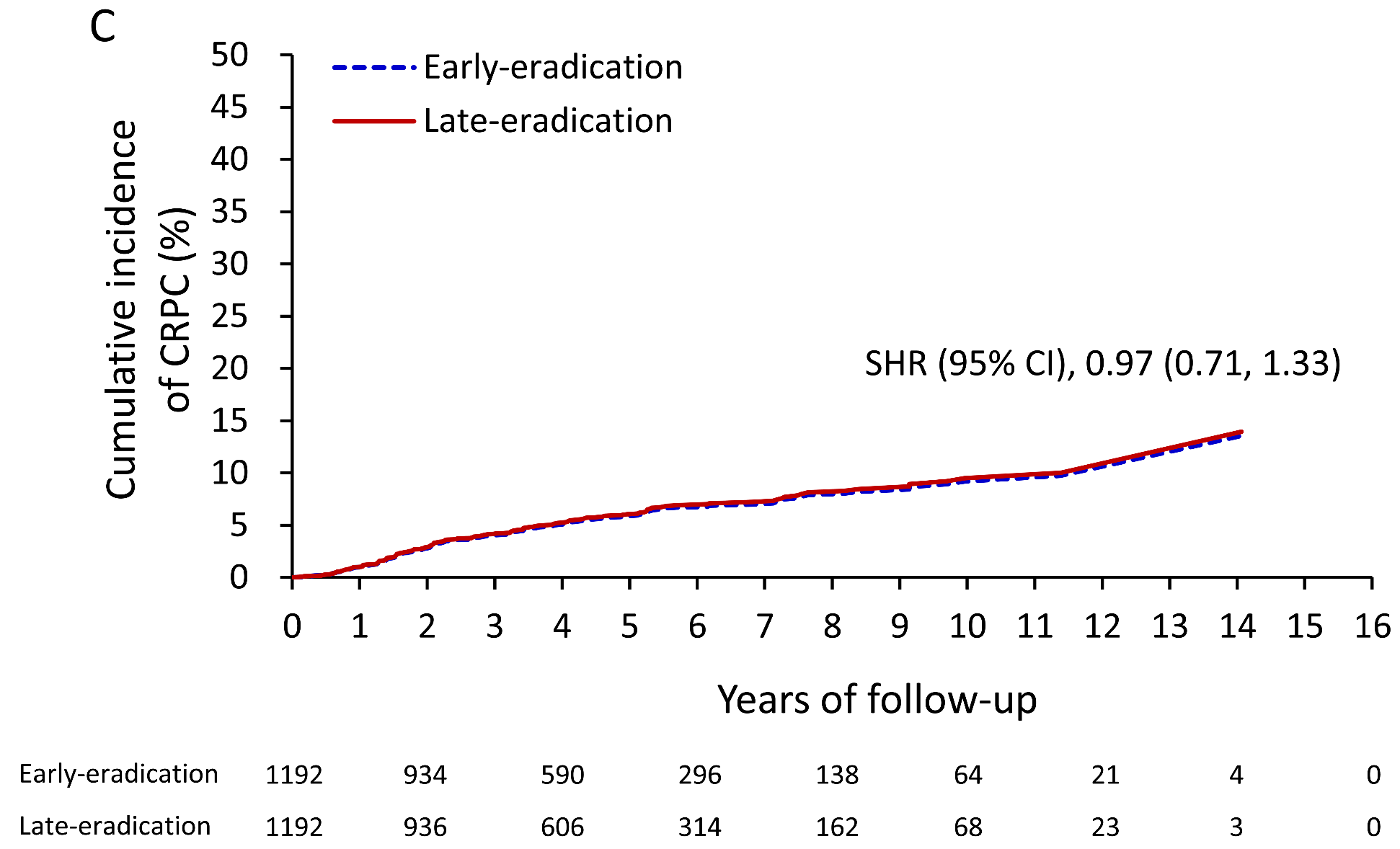


**References**

1. Cheng TM. Reflections on the 20th anniversary of Taiwan's single-payer National Health Insurance System. *Health Aff (Millwood)*. 2015;**34**:502-510.

2. American Hospital Association, American Medical Record Association, Health Care Financing Administration, Statistics NCfH. ICD-9-CM coding and reporting official guidelines. American Hospital Association, American Medical Record Association, Health Care Financing Administration, National Center for Health Statistics. *Journal (American Medical Record Association)*1990 ;**61**:suppl 1-17.

3. World Health Organization. ICD-10 : international statistical classification of diseases and related health problems : tenth revision, 2nd ed. 2004. World Health Organization.

4. Chang SS, Hu HY. Helicobacter pylori Eradication within 120 Days Is Associated with Decreased Complicated Recurrent Peptic Ulcers in Peptic Ulcer Bleeding Patients. *Gut Liver.* 2015;9:346-352

5. Tritchler D. Interpreting the standardized difference. *Biometrics*. 1995;**51**:351-353.

6. Fine JP, Gray RJ. A proportional hazards model for the subdistribution of a competing risk. *J Am Stat Assoc*.1999; **94**:496–509.
